# Latest impact of engineered human liver platforms on drug development

**DOI:** 10.1063/5.0051765

**Published:** 2021-07-16

**Authors:** Chase P. Monckton, Grace E. Brown, Salman R. Khetani

**Affiliations:** Department of Bioengineering, University of Illinois at Chicago, Chicago, Illinois 60607, USA

## Abstract

Drug-induced liver injury (DILI) is a leading cause of drug attrition, which is partly due to differences between preclinical animals and humans in metabolic pathways. Therefore, *in vitro* human liver models are utilized in biopharmaceutical practice to mitigate DILI risk and assess related mechanisms of drug transport and metabolism. However, liver cells lose phenotypic functions within 1–3 days in two-dimensional monocultures on collagen-coated polystyrene/glass, which precludes their use to model the chronic effects of drugs and disease stimuli. To mitigate such a limitation, bioengineers have adapted tools from the semiconductor industry and additive manufacturing to precisely control the microenvironment of liver cells. Such tools have led to the fabrication of advanced two-dimensional and three-dimensional human liver platforms for different throughput needs and assay endpoints (e.g., micropatterned cocultures, spheroids, organoids, bioprinted tissues, and microfluidic devices); such platforms have significantly enhanced liver functions closer to physiologic levels and improved functional lifetime to >4 weeks, which has translated to higher sensitivity for predicting drug outcomes and enabling modeling of diseased phenotypes for novel drug discovery. Here, we focus on commercialized engineered liver platforms and case studies from the biopharmaceutical industry showcasing their impact on drug development. We also discuss emerging multi-organ microfluidic devices containing a liver compartment that allow modeling of inter-tissue crosstalk following drug exposure. Finally, we end with key requirements for engineered liver platforms to become routine fixtures in the biopharmaceutical industry toward reducing animal usage and providing patients with safe and efficacious drugs with unprecedented speed and reduced cost.

NOMENCLATURE

BOC: body-on-a-chip
BSEP: bile salt export pump
CRISPR: clustered regularly interspaced short palindromic repeats
CYP: cytochrome P450 enzymes
DDIs: drug–drug interactions
DILI: drug-induced liver injury
DMEs: drug metabolizing enzymes
ECM: extracellular matrix
FDA: Food and Drug Administration
GelMA: gelatin methacryloyl
HBV: hepatitis B virus
HCV: hepatitis C virus
HMVEC: human microvascular endothelial cells
HSCs: hepatic stellate cells
HTS: high-throughput screening
iHeps: iPSC-derived hepatocyte-like cells
iPSC: induced-pluripotent stem cell
KCs: Kupffer cells
LAMPS: liver acinus microphysiological system
LPS: lipopolysaccharide
LSECs: liver sinusoidal endothelial cells
MOS: margin of safety
MPCC: micropatterned coculture
MPOC: metabolic patterning on a chip
MPS: microphysiological system
MRP2: multidrug resistance-associate protein 2
NAFLD: nonalcoholic fatty liver disease
NPCs: non-parenchymal cells
NTCP: sodium taurocholate co-transporting polypeptide
PHHs: primary human hepatocytes
PKPD: pharmacokinetics and pharmacodynamics
TEER: transepithelial/transendothelial electrical resistance
THLE: transformed human liver epithelial
UGT: UDP glucuronosyltransferase

## INTRODUCTION

**FIG 1.**
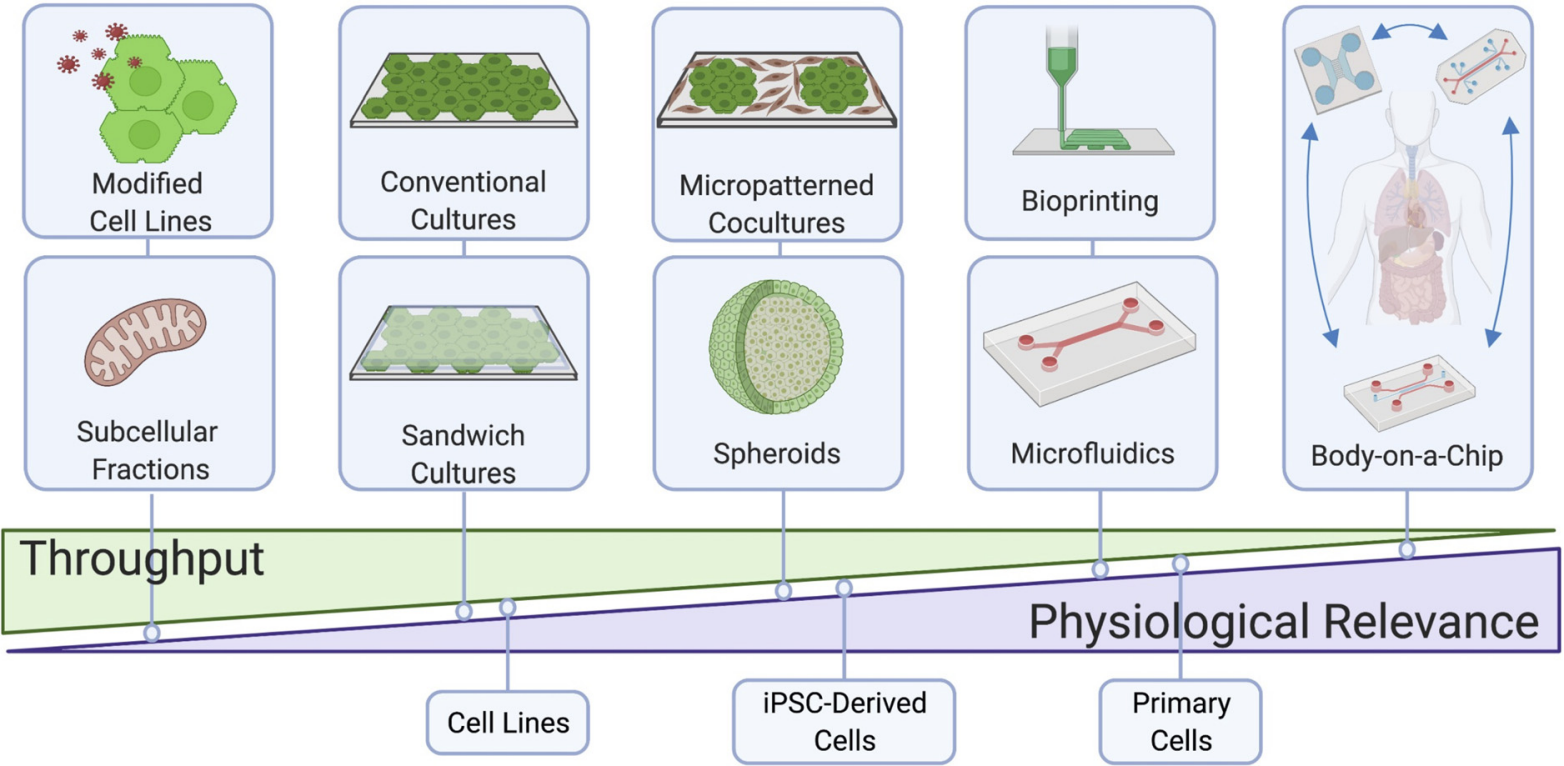
Commercially available liver models with different throughput, technological and physiological complexities, and cell sources for drug development. Figure created using BioRender.

The process of drug development takes 12–15 years and $3–5 billion to bring a single drug to the market.^1,2^ Thus, the withdrawal of a drug from the marketplace comes with a tremendous cost to the pharmaceutical/biotech industry and the economy; more importantly, drug withdrawal deprives patients of potentially life-saving therapies for chronic diseases. Drugs are often withdrawn due to severe adverse reactions and of such, ∼25% of drug withdrawals^3^ as well as 22% of black-box warnings on marketed drugs^4^ are due to drug-induced liver injury (DILI). Therefore, it is highly desirable to eliminate drugs that have the highest risk to cause human DILI earlier in the drug development pipeline prior to live patient exposure. However, significant differences across species in liver metabolic pathways necessitate the use of *in vitro* models of the human liver during preclinical drug testing to mitigate DILI risk.^5,6^ Primary human liver cells are ideal for fabricating such models given their physiological relevance; however, these cells rapidly lose their phenotypic functions within conventional 2-dimensional (2D) monoculture formats.^7,8^ Such issues can be mitigated via the use of bioengineering tools that allow recapitulation of key physiological cues *in vitro* to the extent necessary for different throughput needs within the various stages of drug development (Fig. 1). Here, we will showcase the design features of those engineered *in vitro* liver models that have impacted the drug development pipeline for different applications. We will primarily focus on models that have entered the commercial landscape and been utilized by the biopharmaceutical industry in specific case studies, while also discussing emerging next generation models that are nearing deployment to the marketplace, including dual-organ and body-on-a-chip (BOC) devices useful to understand how metabolism of drugs by the liver affects other organs and vice versa. We will end with a discussion on key considerations for emerging liver and multi-organ platforms to be adopted by the biopharmaceutical industry for routine drug screening.

## SCREENING FOR LIVER LIABILITY DURING EARLY DRUG DEVELOPMENT

Screening of large compound libraries, ranging from 10^6^ to 10^7^ compounds,^9^ for efficacy and toxicity is too costly and time-consuming to perform in live animals. Therefore, high-throughput screening (HTS) *in vitro* platforms are required for the identification of lead compounds from large compound libraries. In the case of the liver, the simplest models used for drug discovery are isolated subcellular fractions, such as microsomes containing drug metabolizing enzymes (DMEs)^10^ or isolated mitochondria.^11^ To this end, mitochondrial liability, which is observed in ∼50% of drugs labeled with black box warnings,^12,13^ was used to predict the toxicity of known DILI compounds with 92% sensitivity; however, 36% of non-DILI compounds were falsely predicted as toxic (i.e., low specificity). Therefore, cell-based models are required to replicate higher-order drug processes [i.e., biotransformation, secondary metabolite formation, transporter kinetics, and drug–drug interactions (DDIs)] to enable both high sensitivity and specificity.

Cell lines, such as SV-40 transformed human liver epithelial (THLE) and cancerous HepG2, have been previously used to predict DILI as they are inexpensive and easily expandable for HTS. For example, galactose, substituted for glucose, was used to force mitochondrial oxidative phosphorylation and improve the ability of HepG2 cells to predict toxicity *in vitro*.^14,15^ THLE cells, which can be easily transfected with cytochrome P450 (CYP) enzymes, such as CYP3A4, were adapted to 384-well plates and demonstrated a positive predictive value of 91% and negative predictive value of 21%.^16^ Subcellular fractions and modified cell lines have been utilized in combination to improve DILI prediction outcomes. For instance, AstraZeneca combined drug-mediated THLE toxicity, THLE-3A4 toxicity, HepG2 mitochondrial injury, inhibition of bile salt export pump (BSEP), inhibition of multidrug resistance-associate protein 2 (MRP2), and drug covalent binding to human hepatocytes to achieve 100% sensitivity and 78% specificity for 27 drugs with known DILI liability.^17^ Additionally, Pfizer also demonstrated that cell viability assays, damage to mitochondria, and clinical C_max_ (the maximum concentration of drug observed in human plasma) values of greater than 1 *μ*M were most predictive of drug-induced hepatotoxicity outcomes.^18^ We have recently shown that HepaRG, a cancerous cell line that has the potential to differentiate into either hepatocytes or cholangiocytes (bile ductal cells), benefits from coculture with murine embryonic fibroblasts in both 2D and 3-dimensional (3D) formats with respect to higher metabolic functions and improved sensitivity of 54% (and specificity of 100%) for DILI detection than HepaRG monocultures (∼16% sensitivity).^19^ However, while cell lines allow for HTS, they grow abnormally and have significantly lower DME and transporter activities as evident in a proteomics analysis which noted a 90% reduction of CYP enzymes in HepG2 cells as well as a decrease in drug transporter expression relative to primary human hepatocytes (PHHs).^20^ Similarly, while HepaRG/fibroblast cocultures showed improved DILI detection as discussed above, the sensitivity was still significantly lower than PHH/fibroblast cocultures (∼70%–75%) treated with the same drug set.^6^ Nonetheless, since cell lines have high specificity for DILI detection, they are still useful for a tier 1 screen to eliminate the most toxic compounds from further development.

Given the above limitations with transformed cell lines, PHHs are preferred for drug screening at later stages of drug development when the number of compounds has been winnowed down to <100. PHHs are routinely used in suspension cultures to evaluate drug clearance and drug metabolite formation; however, these incubations are limited to 4–6 h since PHHs are an adherent cell type, and thus, PHH suspensions are useful for only evaluating compounds with high expected turnover *in vivo*.^21^ PHHs can attach to hard surfaces (e.g., polystyrene or glass) coated with adsorbed collagen-I; however, in this conventional 2D format, PHHs display a precipitous decline in DME and transporter activities, which makes it difficult to obtain clinically relevant results for drug transport, metabolism, and toxicity.^7,8^ To slow down such a rapid decline in phenotypic functions, PHHs can be sandwiched between extracellular matrix (ECM) layers of collagen-I and/or Matrigel^™^, which also has the added benefit of recovering the bile canaliculi between hepatocytes.^22^ For DILI assessment, a landmark study by Pfizer exposed collagen/Matrigel sandwiched PHHs to >300 drugs for 24 h and then assessed several known markers of DILI (mitochondrial membrane potential, reactive oxygen species, and intracellular glutathione); a sensitivity of 50%–60% and specificity of 95%–100% were observed.^23^ However, due to a decline in CYPs in ECM sandwiched hepatocytes,^24,25^ this model is not always suitable for drug treatment for longer than 1–3 days, which is an important determinant of improved sensitivity for DILI detection without compromising specificity.^6^ The limitations with conventional and ECM sandwiched PHH cultures can be mitigated to a great extent with bioengineering tools that we discuss in the section on engineered human liver models validated for drug testing.

## ENGINEERED HUMAN LIVER MODELS VALIDATED FOR DRUG TESTING

Many engineered liver models have been developed over the last 15 years for a variety of applications, ranging from drug metabolism and toxicity screening, disease modeling for the discovery of novel therapeutics, and cell-based therapies (i.e., regenerative medicine). Here, we focus on those engineered human liver models that are commercially available and have been validated to some extent by the biopharmaceutical industry for drug metabolism and toxicity screening, while referring the reader to other review articles in which the use of a larger variety of liver models is discussed for the other applications above.^26,27^

### Micropatterned cocultures (MPCCs)

**FIG. 2.**
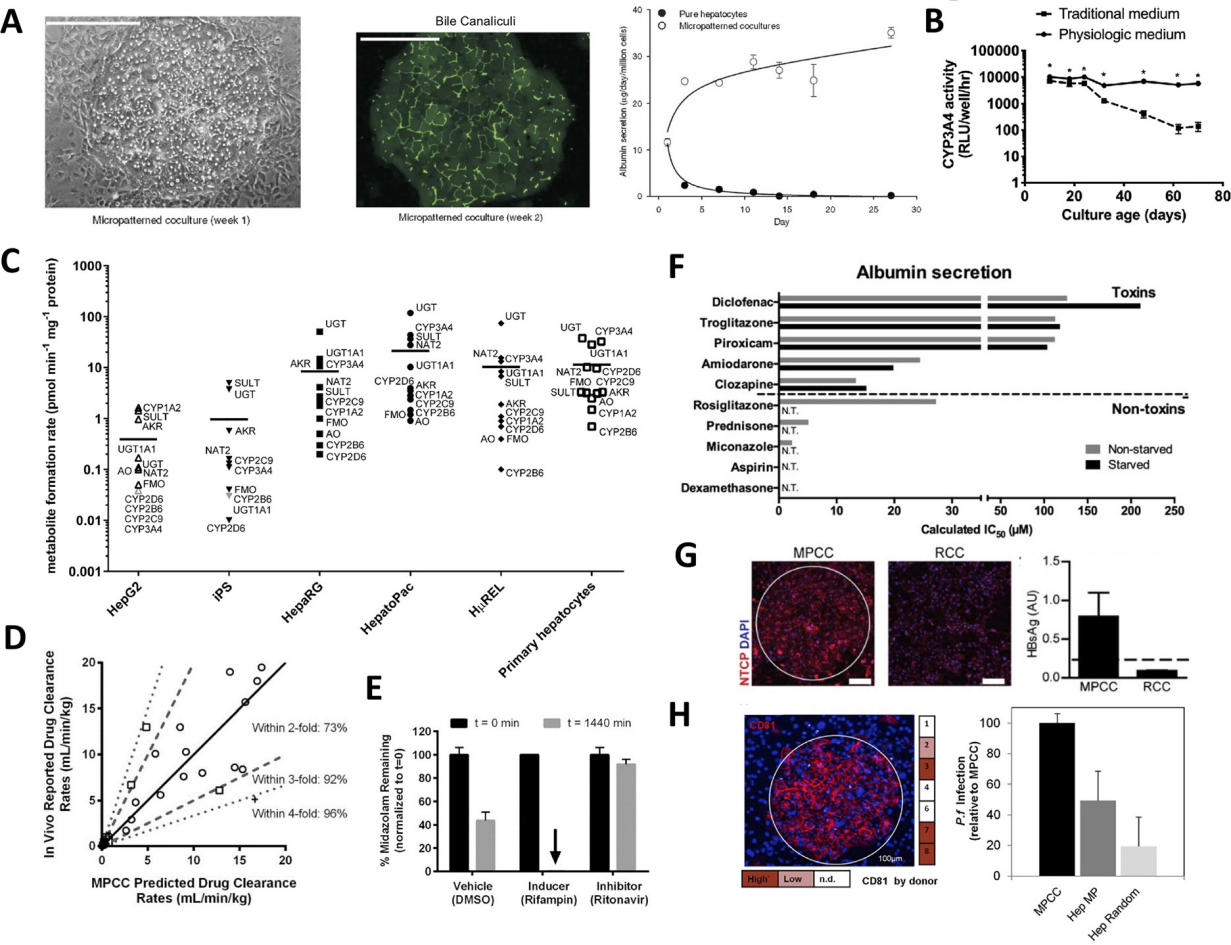
Micropatterned cocultures (MPCCs). (a) Primary human hepatocytes (PHH) display prototypical morphology (left, phase contrast), bile canaliculi formation (middle, transport of fluorescent dye), and relatively stable albumin secretion (right) within micropatterned clusters of empirically optimized dimensions and when surrounded by 3T3-J2 murine embryonic fibroblasts.^7^ Reproduced with permission from Khetani and Bhatia, Nat. Biotechnol. **26**(1), 120 (2008). Copyright 2008 Springer Nature. (b) MPCCs subjected to a physiologically inspired medium containing human serum and physiologic insulin levels improved stability of functions (CYP3A4 shown here) to almost 10 weeks as compared to the use of the traditional (bovine serum, high insulin) culture medium utilized in the field of hepatocyte culture systems for drug screening.^34^ Reprinted with permission from Davidson *et al.*, Toxicology **449**, 152662 (2021). Copyright 2021 Elsevier. (c) Formation of drug metabolites mediated by different CYP enzymes across different culture models, such as HepG2 and HepaRG cancerous cell lines, induced pluripotent stem (iPSC) cell-derived hepatocyte-like monocultures, PHHs in suspension, randomly distributed cocultures of PHHs and 3T3-J2 fibroblasts (H*μ*REL), and MPCCs (commercial name HepatoPac^™^). MPCCs had the highest levels of enzymatic activities overall.^51^ Reprinted with permission from Kratochwil *et al.*, AAPS J. **19**(2), 534–550 (2017). Copyright 2017 Author(s), licensed under a Creative Commons Attribution 4.0 International License. (d) Correlation of clearance rates for 26 compounds (high, medium, and low turnover compounds) obtained from MPCCs and as observed in the clinic.^39^ Reproduced with permission from Lin *et al.*, Drug Metab. Dispos. **44**(1), 127–136 (2016). Copyright 2016 ASPET. (e) Clearance of midazolam, a CYP3A4 substrate, was significantly enhanced when MPCCs were preincubated with CYP3A4 inducer drug, rifampin, and significantly inhibited when MPCCs were preincubated with CYP3A4 inhibitor drug, ritonavir, which is also observed in the clinic in humans.^39^ Reproduced with permission from Lin *et al.*, Drug Metab. Dispos. **44**(1), 127–136 (2016). Copyright 2016 ASPET. (f) Intermittently starving MPCCs of hormones and serum (bovine) every week for 2 days improves functional lifetime and prediction of drug toxicity outcomes as observed with interpolated IC_50_ values for toxins but lack of such values for non-toxins (N.T. = not toxic); on the other hand, non-starved cultures displayed several false positive compounds.^33^ Davidson and Khetani, Toxicol. Sci. **174**(2), 266–277 (2020). Copyright 2020 Oxford University Press. (g) MPCCs display higher levels of sodium taurocholate co-transporting polypeptide (NTCP) as compared to random distributed cocultures (RCCs), which led to higher infectivity with hepatitis B virus (HBV) as evident from increased levels of shed HBV “s” antigen in supernatants (HBsAg).^46^ Reproduced with permission from Shlomai *et al.*, Proc. Natl. Acad. Sci. **111**(33), 12193–12198 (2014). Copyright 2014 Author(s). (h) CD81, a *Plasmodium* entry factor, levels were high for 3 PHH donors cultured in MPCCs (left), which led to higher infection of MPCCs with *P. falciparum* (i.e., malaria) as compared to micropatterned hepatocytes only (Hep MP) or RCCs.^47^ Reprinted with permission from March *et al.*, Cell Host Microbe **14**(1), 104–115 (2013). Copyright 2013 Elsevier.

ECM proteins can be micropatterned onto hard or soft surfaces using soft lithographic techniques, such as elastomeric stamping, stenciling, plasma ablation, printing, and microfluidic patterning.^28,29^ Khetani and Bhatia^7^ developed MPCCs (commercially known as HepatoPac^®^) of hepatocytes micropatterned onto collagen-coated circular domains of empirically optimized dimensions within industry standard multiwell plates (up to 384-well format^30^) and subsequently surrounded by 3T3-J2 murine embryonic fibroblasts, which express molecules present in the liver, such as T-cadherin and decorin.^31,32^ PHHs maintain *in vivo*-like morphology, polarity, and functions for 4 weeks in MPCCs [Fig. 2(a)], and such longevity can be extended to up to 10 weeks by either intermittently starving the cultures of serum and hormones each week for 2 days^33^ or by using a physiologic culture medium containing human serum^34^ [Fig. 2(b)]. Interestingly, 3T3-J2 fibroblasts were found to support primary hepatocyte functionality to the highest levels compared to other 3T3 fibroblast clones,^31^ primary human liver sinusoidal endothelial cells (LSECs),^35^ primary human hepatic stellate cells (HSCs),^36^ and primary human Kupffer cells (KCs), the resident macrophage of the liver.^37^ However, when the above liver non-parenchymal cells (NPCs) were introduced alongside the 3T3-J2 fibroblasts in MPCCs, interactions of functionally stabilized PHHs and liver NPCs could still be modeled in clinically relevant ways as shown in the above-mentioned studies. Furthermore, 3T3-J2 fibroblasts have advantages for use as a PHH-supporting stromal cell since the fibroblasts are expandable, contact-inhibited, and lack liver- and human-specific functions and gene expression, such as CYP enzymes.^7^ The MPCC platform and its variants have been rigorously validated for several applications within drug development, such as prediction of drug clearance,^38,39^ drug metabolite identification across different species,^40,41^ drug–drug interactions (DDIs),^39,42^ DILI prediction,^6,43,44^ infection with hepatitis B/C viruses (HBV/HCV),^45,46^ malaria infection,^47^ and early stages of nonalcoholic fatty liver disease (NAFLD).^36,48,49^ We provide representative examples below from pharmaceutical practice where available.

Major metabolites of drugs need to be identified during preclinical drug testing toward testing their pharmacological and toxic effects prior to the initiation of human clinical trials.^50^ Toward that end, Roche showed that MPCCs displayed higher levels of several DMEs as compared to PHH suspensions, randomly distributed cocultures of the same two cell types (H*μ*REL^™^), HepG2, HepaRG, and induced-pluripotent stem cell (iPSC)-derived hepatocyte monocultures (FujiFilm's iCell^®^) [Fig. 2(c)].^51^ In a separate effort, Pfizer showed that following a 7-day incubation with 27 compounds, MPCCs generated 75%–82% of clinically relevant metabolites whereas PHH suspensions generated 53%–64% following a 4-h incubation that is possible with such cultures.^40^ In a follow-up study, Pfizer showed that primary hepatocytes from different species (human, dog, monkey, and rat) could be used in the MPCC platform to determine similarities and differences in the generation of drug metabolites across species;^41^ such an analysis is particularly useful to determine which animal species to utilize for the rodent and non-rodent live animal studies required by the U.S. Food and Drug Administration (FDA). Finally, Boehringer Ingelheim showed that MPCCs containing PHHs produced clinically relevant levels of metabolites of faldaprevir, a drug used to inhibit a protease of HCV.^52^

The accurate assessment of hepatic drug clearance *in vivo* using *in vitro* assays is important to adequately estimate drug doses in the clinic in humans.^53^ However, clearance of low turnover drugs (with desirable one pill a day dosing regimen) is especially difficult to assess in 4–6 h incubations in suspension PHHs, which can be mitigated with long-term incubations on MPCCs.^54^ For instance, Boehringer Ingelheim showed that long-term incubations on MPCCs could be used to predict the clearance of ten low clearance compounds within an acceptable threshold of threefold of reported clinical values, and that compound turnover was twofold higher in MPCCs than in suspension PHHs.^38^ In a follow-up study, we showed that MPCCs predicted the clearance of 92% of 26 drugs within threefold of their clinical clearance values [Fig. 2(d)], whereas conventional 2D PHH monocultures and PHH suspensions were only 20% predictive and could not metabolize several drugs.^39^ Roche further showed that MPCC is a suitable tool for the estimation of metabolic clearance within an acceptable threshold of two to threefold of clinical values.^55^ Sanofi showed that MPCCs, adjusted for the unbound drug concentration in plasma and the albumin-facilitated drug uptake phenomena, provided better prediction of hepatic clearance than PHH monocultures and PHH suspensions, potentially due to more physiologic amounts of albumin secreted by MPCCs as compared to the conventional models.^56^ Finally, Takeda used MPCCs to study the clearance and metabolism of TAK-041, a drug currently being developed to treat cognitive disorders;^57^ TAK-041 had higher turnover in MPCCs as compared to suspension hepatocytes, and MPCCs provided the ability to elucidate complex biotransformation mechanisms for this compound.

DDIs in the clinic can lead to reduced efficacy or toxicity of one or more co-administered drugs; therefore, the potential for DDIs is typically evaluated during preclinical drug screening via the drug-mediated induction or inhibition of CYP and other hepatic enzymes.^58^ We showed that the clearance of “victim” drugs could be modulated in clinically relevant ways upon incubation with “perpetrator” drugs that are known to either induce or inhibit key CYPs^39^ [Fig. 2(e)]. Boehringer Ingelheim showed that the knockdown of CYP3A4 in MPCCs led to surprising increases in the activities of CYP2C9 and UDP-glucuronosyltransferase (UGT) 2B7 activity.^59^ The same company also showed that erythromycin, an inhibitor of CYP3A4, significantly inhibited in MPCCs the clearance of midazolam and alprazolam, two CYP3A4 substrates.^60^ Finally, Vertex showed that drug-mediated induction of CYP2C enzymes was higher in MPCCs than in PHH monocultures;^61^ furthermore, EC50 values (drug dose that causes 50% of maximal CYP induction) in MPCCs for rifampin-mediated CYP3A4 and CYP2C9 induction were more clinically relevant than monocultures, which was subsequently found to be due to higher uptake transporter activities in MPCCs that leads to more intracellular accumulation of drugs like rifampin than the monocultures, which were shown to have reduced transporter activities.^62^

MPCCs have also been validated for DILI prediction. For instance, Pfizer showed using a panel of 45 drugs that MPCCs with PHHs predicted DILI-positive compounds with a sensitivity of 65.7% using a combination of secreted (albumin and urea) and intracellular [Adenosine Triphosphate (ATP) and glutathione] markers as opposed to 28.6% sensitivity for ECM sandwiched PHHs and 48.6% sensitivity for MPCCs with rat hepatocytes, while all models maintained at least 90% specificity;^6^ importantly, the sensitivity of MPCCs with PHHs for the most liver toxic compounds (i.e., those with black-box warnings or market withdrawals due to DILI) was 100%. A key reason for significant differences in sensitivities for ECM sandwiched PHHs across the above study^6^ and a previous study by Pfizer (50%–60% sensitivity^23^) was the selection of drugs, such that in the former study,^6^ more false negative drugs (i.e., hepatotoxic in the clinic but non-hepatotoxic *in vitro*) in ECM sandwiched PHHs were selected (25 out of 35) to determine if MPCCs could detect hepatotoxicity of some or all of the false negatives toward improving Pfizer's internal DILI assessment strategy. More generally, selection of drugs, specific endpoints, and decision algorithm (e.g., percent downregulation of end point to call a compound as “toxic” *in vitro*) can affect the sensitivity and/or specificity of a platform in a particular study.

Otsuka Pharmaceuticals showed that tolvaptan, a drug used to treat hyponatremia associated with different diseases, induced cellular stress and exosomal release of microRNA-122 from MPCCs, which may be a precursor to idiosyncratic DILI caused by tolvaptan due to the activation of the adaptive immune system.^63^ Merck recently showed using a panel of 93 compounds that MPCCs can be used with a key transcriptomic signature to detect DILI-positive compounds with 68% sensitivity and 86% specificity in a resource-sparing and higher throughput approach.^43^ We showed that fialuridine, a nucleoside analog for HBV infection and a severe liver toxin, was toxic to MPCCs with PHHs upon repeat treatment, but not those with rat hepatocytes.^64^ We also showed that intermittently starved MPCCs displayed higher specificity (low false positives) for DILI detection even after 5 weeks of culture as compared to non-starved (traditional) MPCCs [Fig. 2(f)]. Finally, when using iPSC-derived human hepatocyte-like cells (iHeps) in MPCCs, sensitivity for DILI detection was found to be 65% vs 70% for MPCCs with PHHs, while specificity was 100% for both models;^65^ these results suggest that MPCCs with iHeps can provide a potential path for precision (patient-specific) drug testing with a large number of iPSC lines to elucidate the role of genetics in DILI.

MPCCs have also been shown to be useful for disease modeling, including long-term infection and replication of HBV,^46^ HCV,^45^ and malaria.^47^ Roche showed that 30% of PHHs in MPCCs could be infected with patient-derived HBV.^42^ MPCCs were also shown to have higher replication of HCV^45^ and HBV^46^ than randomly distributed cocultures of the same two cell types infected with the same viral stocks; in the case of HBV, the differences in infectivity in MPCCs vs random cocultures are likely due to higher levels of a receptor for HBV, the sodium taurocholate co-transporting polypeptide (NTCP)^46^ [Fig. 2(g)]. MPCCs can recapitulate the full liver stage of parasites that cause malaria, *Plasmodium falciparum* and *Plasmodium vivax*, including the release of infected merozoites and infection of overlaid erythrocytes, and also the establishment of small forms in late liver stages of *P. vivax*.^47^ Infected MPCCs were able to recapitulate the known effects of antimalarial drugs [Fig. 2(h)], and anti-hypnozoite candidate compounds have been successfully tested on MPCCs infected with *P. vivax*.^30^ Finally, Merck augmented MPCCs with KCs to investigate the effects of exogenous proinflammatory cytokines or those secreted by lipopolysaccharide (LPS)-activated KCs on hepatic CYP expression and activity as in inflammation and infection *in vivo.*^37^

Unlike ECM sandwiched PHHs, gluconeogenesis is retained in MPCCs for several weeks and can be modulated in physiologically relevant ways with pancreatic hormones, insulin and glucagon;^49^ such a capability allowed for the demonstration of clinically relevant efficacy of metformin, a drug used for type 2 diabetes mellitus, in downregulating gluconeogenesis without causing toxicity at the dose range tested. Finally, we showed that MPCCs augmented with activated (myofibroblastic) HSCs displayed the early stages of nonalcoholic fatty liver disease (NAFLD) with steatosis in PHHs, downregulation of CYP enzymes and transporters, and collagen-I deposition by HSCs; this model system was shown to be amenable to screening of clinically relevant compounds and their combinations on alleviating the hepatic dysfunctions in NAFLD.^36^

### Three-dimensional (3D) spheroids, organoids, and bioprinted tissues

**FIG. 3.**
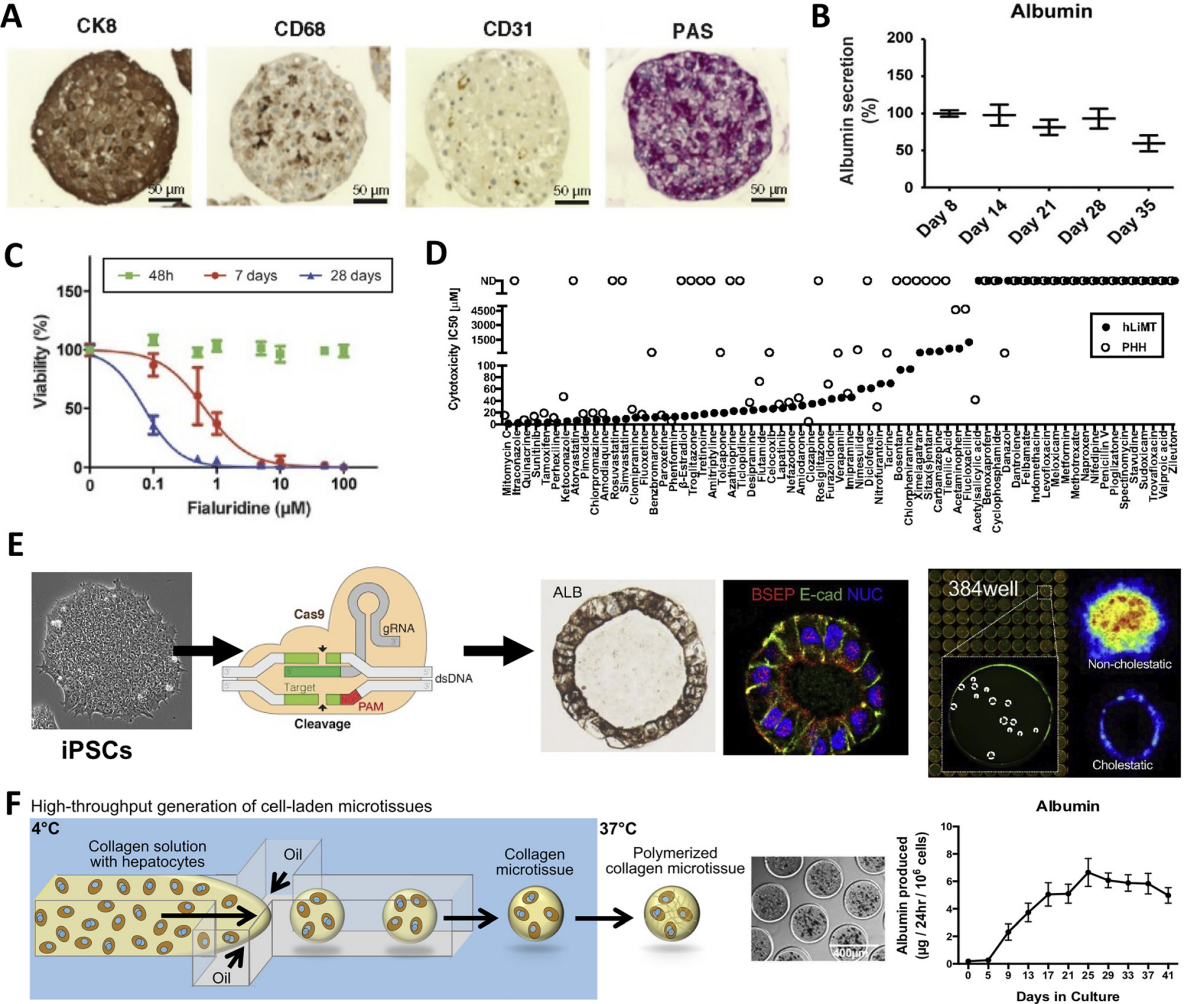
3D spheroids, organoids, and bioprinted liver tissues. (a) InSphero spheroids containing primary human hepatocytes (PHHs, stained for glycogen with period acid Schiff, PAS, stain and positive for CK8), liver sinusoidal endothelial cells (CD31 positive), and Kupffer cells (CD68 positive).^67^ Reprinted with permission from Messner *et al.*, Arch. Toxicol. **87**(1), 209–213 (2013). Copyright 2013 Author(s), licensed under a Creative Commons (CC-BY) license. (b) Long-term retention of liver function (albumin shown here) in PHH-containing spheroids generated using commercially available ultra-low attachment plates.^70^ Reprinted with permission from Bell *et al.*, Sci. Rep. **6**, 25187 (2016). Copyright 2016 Author(s), licensed under a Creative Commons Attribution (CC BY) license. (c) Viability assessment via cellular ATP content of fialuridine-induced toxicity was improved over long-term (i.e., 28 days) treatment every 48-h in PHH-containing spheroids generated using ultra-low attachment plates.^70^ Reprinted with permission from Bell *et al.*, Sci. Rep. **6**, 25187 (2016). Copyright 2016 Author(s), licensed under a Creative Commons Attribution (CC BY) license. (d) A panel for drug compounds classified as severe, high, or low clinical drug-induced liver injury (DILI) positive were compared in InSphero PHH-containing spheroids (human liver microtissues or hLiMT) and 2D PHH monocultures; hLiMT predicted more IC_50_ values than 2D monocultures.^73^ Reprinted with permission from Proctor *et al.*, Arch. Toxicol. **91**(8), 2849–2863 (2017). Copyright 2017 Author(s), licensed under a Creative Commons (CC-BY) license. (e) Left to right: iPSCs, derived from diverse genetic backgrounds can be further gene edited using CRISPR. These iPSCs are then differentiated into foregut cells (not shown) in 2D monolayers, detached, and differentiated into human liver organoids (HLOs) with lumens in 384-well plates.^84^ High content imaging can be conducted on HLOs to determine the effects of drugs on markers, such as fluorescent bile analog excretion into the HLO lumen. Reprinted with permission from Shinozawa *et al.*, Gastroenterology **160**(3), 831–846 (2021). Copyright 2021 Elsevier. (f) A high-throughput droplet microfluidic device for the generation of 3D liver microtissues.^85^ Left to right: Hepatocytes are suspended in collagen solution and perfused through the microfluidic device at 4 °C (to keep collagen from polymerizing) with an oil stream; since oil and water do not mix, the collagen + cells form spherical droplets. These so-called microtissues are collected, heated at 37 °C to promote collagen polymerization and cell encapsulation, oil is drained, and microtissues are resuspended in culture medium within microwells in static or fluidic plates/devices. The hepatocytes can be cocultured with non-parenchymal cell (NPC) types by either co-encapsulating both cell types within the microtissue or by seeding/coating the NPCs onto the surface of the polymerized collagen-based hepatic microtissues. The PHH microtissues coated with 3T3-J2 fibroblasts display stable liver functions (albumin shown here) for at least 6 weeks *in vitro*. Adapted with permission from Kukla *et al.*, Gene Expression **20**(1), 1 (2020). Copyright 2020 Author(s), licensed under a Creative Commons Attribution-NonCommercial-NoDerivatives 4.0 International (CC BY-NC-ND 4.0) license.

3D liver models are known to recapitulate cell–cell and cell–ECM interactions and stabilize liver functions better than conventional 2D monocultures.^66^ PHHs and liver NPCs can be aggregated into spheroids using a hanging-drop plate-based method developed by InSphero [Fig. 3(a)], which displayed some hepatic functions (e.g., albumin secretion and glycogen storage) for 5 weeks.^67^ More broadly, 97.5% of mRNA transcripts and 92.7% of proteins were found to be relatively stable in PHH/NPC spheroids over 35 days in culture as assessed via DNA microarrays and proteomics, respectively;^68^ however, the transcriptome diverged over 2 weeks in culture as compared to the day of spheroid formation, but was still more closely related to human liver tissue than HepaRG, HepG2, iHeps, and even liver slices.^69^ Spheroids can also be formed in commercially available ultra-low attachment plates (e.g., Corning, Inc.) with long-term stability of liver functions [Fig. 3(b)].^70^ A multicenter study showed long-term stability of protein expression and increased levels of DME and transporter proteins in such PHH spheroids as compared with ECM sandwiched PHHs.^71^

Liver spheroids have been validated for DILI detection and drug-mediated CYP induction studies. For instance, fialuridine was toxic to PHH spheroids upon repeat treatment [Fig. 3(c)], which was mitigated by inhibiting the expression or activity of proteins implicated in mitochondrial transport and activation of fialuridine via phosphorylation.^70^ In a more comprehensive study, when PHH spheroids were treated with 123 drugs and evaluated for intracellular ATP, sensitivity was 69% and specificity was 100%.^72^ Similarly, Genentech used a panel of 110 drugs and a margin of safety (MOS = IC_50_/C_max_, where IC_50_ is the concentration that inhibits measured end point by 50%) of 50× to show that InSphero spheroids predicted DILI with a 52.2% sensitivity vs 33.3% in 2D culture and specificity was 85.4% in both models [Fig. 3(d)].^73^ In another study, DILI sensitivity was improved in PHH spheroids to 61% as compared to 26% in PHH monocultures when using a MOS of 50× for a panel of 100 drugs; however, specificity was 79% in spheroids vs 100% in monocultures.^74^ Finally, spheroids were found to be useful for assessing drug-mediated CYP3A4 induction, whereas 2D monocultures did not show CYP3A4 induction with some of the prototypical compounds.^75^

Some disease states can be established in spheroids to assess the mechanisms and evaluate the potential drug therapies. HepaPredict^™^ (PHH/NPC spheroids assembled via ultra-low attachment plates) replicated the features of NAFLD (e.g., steatosis and insulin resistance) when treated with lipogenic media for downstream testing of anti-steatotic drugs.^76^ Similarly, PHH-NPC spheroids challenged with fatty acids displayed a NAFLD-like phenotype *in vitro* and were useful to determine the attenuation of fibrotic markers using prototypical drug therapies.^77^ Additionally, AstraZeneca showed that the genetic variant TM6SF2 E167K, previously associated with the increased risk for NAFLD, increased steatosis in PHHs within spheroids by reducing the secretion of apolipoprotein B particles as compared to wild-type control spheroids.^78^ In another study, spheroids containing PHHs, human umbilical vein endothelial cells (HUVECs), KCs, and HSCs were encapsulated in gelatin methacryloyl (GelMA) and treated with fatty acids to model the natural progression of NAFLD and fibrosis; incorporation of HSCs led to elevated inflammatory responses and ECM remodeling.^79^ Other spheroid models have been shown to support HCV^80^ and malaria^81^ infections.

In contrast to spheroids, organoids are typically derived from stem cells, such as iPSCs, and have more complex architectures, including nascent vasculature and lumens.^82,83^ In a recent example, iPSCs were differentiated into foregut cells in 2D monolayers, detached, and placed within Matrigel to initiate organoid formation within 384-well plates using the introduction of key growth factors in the medium at different time-points [Fig. 3(e)].^84^ These organoids contained 75% hepatocyte-like cells and 25% NPCs, displayed higher DME and transporter activities than 2D cultures, and secreted bile acids into their lumens. When incubated with 238 drugs and evaluated for cholestasis (inhibition of secretion of fluorescent bile acid analog into organoid lumens) and intracellular ATP levels, the organoids displayed a sensitivity of 69% and specificity of 100%. Furthermore, the organoids were amenable to the use of iPSC lines with different CYP polymorphisms to determine the impact on drug metabolism and toxicity, and could be gene edited via CRISPR (clustered regularly interspaced short palindromic repeats) technology to determine the role of genotype–phenotype relationships on drug effects.

While self-assembled spheroids and organoids are useful for drug screening as discussed above, they suffer from random distribution of cells and spheroids, in particular, are difficult to form with >50% of PHH lots.^70^ Innovations in 3D tissue fabrication, including novel bioprinting approaches and droplet microfluidics, can be used to mitigate the above limitations with self-assembled spheroids/organoids. For instance, a high-throughput droplet microfluidic technology was used to encapsulate PHHs within ECM microgels, which were subsequently “coated” with 3T3-J2 fibroblasts or HSCs [Fig. 3(f)];^85^ the PHH/fibroblast “microtissues” displayed high levels of liver functions for 6+ weeks *in vitro* and functionally outperformed self-assembled spheroids and macrogels of the same cellular composition. These microtissues displayed clinically relevant CYP induction with prototypical compounds and could distinguish troglitazone toxicity from that of its non-hepatotoxic structural analog drug, rosiglitazone. Additionally, 3D bioprinting allows for precise positioning of liver cells.^86^ Photopolymerization and stereolithography were used to rapidly print iPSC-derived hepatocytes, endothelial cells, and mesenchymal stromal cells into gelatin methacrylate structures;^87^ hepatocyte gene expression and functions were retained for 3 weeks. Alternatively, bioprinted liver tissues fabricated using the NovoGen Bioprinter platform with NovoGel 2.0 “bio-ink” by Organovo sustained long-term functions of PHHs, HSCs, and endothelial cells.^88^ The Organovo model was able to display fibrogenesis due to the proliferation and activation of HSCs following transforming growth factor beta 1 treatment as well as increased trovafloxacin-induced hepatotoxicity upon LPS-mediated KC activation.^37^ More recently, KCs were incorporated into 3D bioprinted liver tissues to assess their role in fibrogenesis;^89^ the release of injury-related markers could be detected over 28 days and demonstrated differences with or without KCs in the model. Finally, microfluidics can be interfaced with 3D printed liver tissues to control fluid shear stress and nutrient transport to the tissues.^90^

### Microfluidic (liver-on-a-chip) systems

Microfabrication tools (i.e., soft-lithography and 3D bioprinting) can be used for constructing microfluidic devices that are useful to subject cells to fluid shear forces and gradients of soluble factors as *in vivo*, while enabling automated delivery of nutrients and removal of waste products.^28^ Polydimethylsiloxane (PDMS) is widely used for microfluidic device fabrication since it is biocompatible, optically transparent, relatively inexpensive, and amenable to rapid prototyping; however, for drug screening applications, the hydrophobic and porous properties of PDMS can cause absorption of certain lipophilic drugs and media additives.^91^ Thus, coating modifications for PDMS or thermoplastic alternatives are being explored for next generation microfluidic devices for cell culture in the drug development pipeline.^92^

**FIG. 4.**
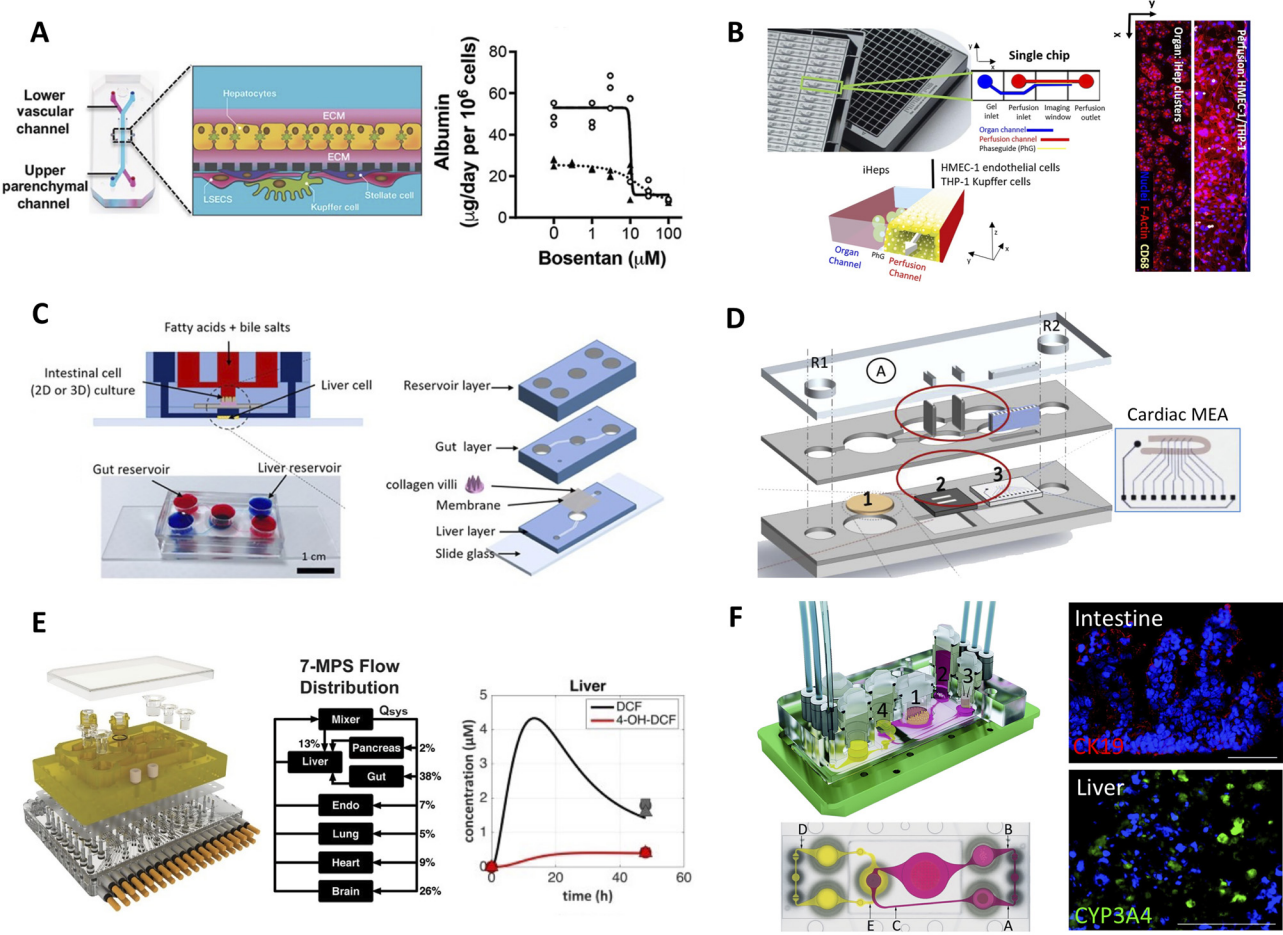
Liver-on-a-chip (microfluidic) platforms. (a) Emulate's liver-chip containing two fluidic channels separated by a porous membrane; ECM sandwiched PHHs are seeded on one side of the membrane while endothelial cells are seeded on the other side. KCs and HSCs can be added optionally to the endothelial side. Right: bosentan toxicity to PHHs in the liver-chip (solid line) and in static ECM sandwiched PHHs (dashed line).^94^ Reprinted with permission from Jang *et al.*, Sci. Transl. Med. **11**(517), eaax5516 (2019). Copyright 2019 AAAS. (b) Mimetas' OrganoPlate with 96 individual chips with hepatocytes seeded in the static channel and human microvascular endothelial cells (HMVEC-1) and THP-1 (monocyte line that can be differentiated into macrophages) seeded in the perfusion channel. Cell presence was verified with F-actin and THP-1 presence was verified with CD68 staining.^108^ Reprinted with permission from Bircsak *et al.*, Toxicology **450**, 152667 (2021). Copyright 2021 Author(s), licensed under a Creative Commons (CC-BY) license. (c) Gut–liver platform allowing for the study of fatty acid metabolism.^118^ Reproduced with permission from Lee *et al.*, Biotechnol. Bioeng. **115**(11), 2817–2827 (2018). Copyright 2018 Wiley. (d) Pumpless microfluidic heart-liver platform with on-chip monitoring of cardiac electrical and mechanical variations. Two laser cut acrylic (top and bottom) layers sandwich two laser cut PDMS layers with PHHs cultured on glass coverslip in chamber 1 and cardiomyocytes cultured on the cantilever array (chamber 2) as well as on the multi-electrode array (MEA, chamber 3). Medium exchange is performed through reservoirs, R1 and R2.^125^ Reprinted with permission from Oleaga *et al.*, Biomaterials **182**, 176–190 (2018). Copyright 2018 Elsevier. (e) Multi-MPS platform allowing for seven interconnected organ systems; each organ model on a Transwell inset can be placed into the configurable device. Right: liver compartment can metabolize diclofenac into its main metabolite, 4-OH-DCF, over time.^128^ Reprinted with permission from Edington *et al.*, Sci. Rep. **8**(1), 4530 (2018). Copyright 2018 Author(s), licensed under a Creative Commons Attribution (CC BY) license. (f) Multi-organ system containing small intestine (compartment 1 in schematic), liver (compartment 2), skin (compartment 3), and kidney (compartment 4) tissue models. The PDMS-glass chip in the device accommodates a surrogate blood flow circuit (pink) and excretory flow circuit (yellow) as also shown in the top view of the device (a–e indicates measurement spots in the flow circuits). Images on the right show microvilli formation (CK19 staining) in the intestine compartment and CYP3A4 in the liver compartment.^131^ Reprinted with permission from Maschmeyer *et al.*, Lab Chip **15**(12), 2688–2699 (2015). Copyright 2015 Author(s), licensed under a Creative Commons Attribution (CC BY 3.0) license.

Several companies have commercialized microfluidic chips for liver cell culture. For example, the H*μ*REL biochip is a device fabricated with four individual polystyrene chips with cellular compartments containing 2D PHH ± NPC cultures interconnected with fluid flow in a polycarbonate housing with specialized tubing;^93^ utilization of these materials leads to decreased drug binding to the device and tubing as compared to conventionally utilized materials. Emulate has marketed the Liver-Chip^™^, a two-channel PDMS-based device separated by a porous membrane and comprised of ECM sandwiched PHHs in the upper compartment, while 2D endothelial layer in the lower compartment [Fig. 4(a)].^94^ HSCs and KCs can be incorporated into the lower channel without compromising hepatic functions, and this model has been reproduced in multiple species to determine species-specific drug metabolism and toxicity differences. In another example, a liver microphysiological system (MPS) commercialized by CN-Bio is composed of 12 individual bioreactors perfused with integrated pumps in a plate-like format, with each bioreactor containing porous scaffolds with PHH ± NPC spheroids attached to the walls of the pores.^95^ This liver MPS with PHHs and multiple donors of KCs was assessed at independent facilities to demonstrate utility for drug metabolism studies.^96^ Consistent increases in lactase dehydrogenase (LDH) release was observed from the synergies of LPS stimulation and trovafloxacin treatment. Importantly, the MPS maintained PHH functions (i.e., albumin secretion was comparable to *in vivo* levels at day 15 of culture) and CYP3A4 activity, which declined in spheroid and ECM-sandwich PHH controls; thus, better resistance to the toxicities of troglitazone and tamoxifen was observed in the MPS.

Liver zonation is the compartmentalization of hepatic and NPC functions along the liver sinusoid due to gradients of oxygen, hormones, nutrients, localized NPC sections, and intestinally derived factors in portal blood.^97^ Due to such gradients, zone-specific drug toxicity is challenging to predict using static *in vitro* liver models;^97,98^ thus, perfusion devices are necessary for such purposes. In the “Liver Acinus MicroPhysiology System” (LAMPS)—a single channel PDMS microfluidic device containing ECM sandwiched PHHs layered with endothelial cells, HSCs, and KCs—variable rates of culture medium flow were used to subject the cells to oxygen tensions from 13% to 3% to recapitulate the tensions in the liver sinusoid;^99^ PHHs subjected to periportal (higher) oxygen tensions displayed higher oxidative phosphorylation, albumin production, and urea secretion while those subjected to pericentral (lower) oxygen tensions displayed higher levels of glycolysis and DMEs (e.g., CYP2E1). Importantly, increased acetaminophen toxicity was observed in pericentral hepatocytes. The Metabolic Patterning on a Chip (MPOC) platform used microfluidic gradient generators to generate gradients of glucagon (high levels induce a periportal phenotype) and insulin (high levels in pericentral region) on 2D PHH monocultures.^100^ Recapitulation of *in vivo*-like nitrogen, carbohydrate, and xenobiotic metabolism was supported, and the MPOC could detect zone-specific toxicity of acetaminophen to the PHH monocultures. Finally, inkjet printing was utilized to fabricate oxygen sensors to allow for real-time monitoring of cellular oxygen consumption in a two-channeled device with 2D PHH monocultures seeded in the bottom chamber and the porous membrane with integrated oxygen sensors placed above the PHHs; these sensors were used to demonstrate a ∼32.5% difference in oxygen consumption across the length of the device.^101^

Microfluidic devices have also been applied to disease modeling. The MPOC above was used to model different degrees of steatosis as in NAFLD in hepatocytes by subjecting them to gradients of free fatty acids.^102^ The device was treated with high levels of oleic and palmitic acid in the culture medium, which led to the accumulation of intracellular triglycerides, upregulation in key genes including CYP2E1 and CYP7A1, and increases in key proteins linked to NAFLD and inflammation; treatment with drugs, such as pioglitazone and metformin, caused a decrease in intracellular triglyceride levels.^103^ In another study, organoids containing iPSC-derived hepatic progenitor cells within micropillar arrays were perfused with fatty acids and as a result displayed lipid accumulation, upregulation of lipid metabolism related genes, and production of reactive oxygen species as in NAFLD.^104^ Finally, microfluidic devices have also been utilized to model HBV infection. For example, PHHs within the aforementioned liver MPS device from CN-Bio required 10 000-fold less multiplicity of infection to sustain HBV infection as compared to conventional spheroids and randomly distributed cocultures of PHHs and 3T3-J2 fibroblasts.^105^

Higher throughput screening can be facilitated through integrated fluidic networks within multi-well plates. For instance, the HepaChip^®^ houses 24 independent culture chambers within the footprint of a standard 96-well plate; dielectrophoresis is used to aggregate PHHs and primary human liver endothelial cells into each chamber, which is then perfused continuously and unidirectionally using a custom pump system.^106^ In another study, PHH functions were supported at higher levels than static controls in a 96-microfluidic array (PREDICT-96) developed using thermoplastic materials and an ultra-low volume recirculating system.^107^ Alternatively, the OrganoPlate LiverTox^™^ system housed in a 384-well plate contains 96 individual 2-channel microfluidic devices allowing for iPSC-derived hepatocytes to be seeded in one (static) channel and endothelial cells and macrophages in the other (fluidic) channel [Fig. 4(b)];^108^ the LiverTox system was more sensitive for the detection of troglitazone toxicity than static monocultures. Finally, a high-throughput platform containing 96 microfluidic devices in a thermoplastic culture plate (same footprint as an industry standard 96-well plate) was developed recently by Draper in collaboration with Pfizer; a programmable perfusion system is integrated into the lid of the plate with 192 microfluidic pumps that can individually address each device and a TEER (Transepithelial/transendothelial electrical resistance) measurement system.^109^ Each of the 96 microfluidic devices has two channels separated by a porous membrane to which ECM sandwiched PHHs were attached to one side and endothelial cells to the other side. PHH CYP activity was improved with a low shear stress flow while the endothelial cells were better aligned with a high shear stress flow in the corresponding channels.

## MULTI-ORGAN-ON-A-CHIP APPROACHES

Body-on-a-chip (BOC) systems with multiple tissue types interconnected via perfused culture medium are necessary to appraise the full spectrum of systemic drug effects. While these BOC systems are still in their nascent stages, key technologies and proof-of-concept studies with drug sets have showcased the potential of such platforms to positively impact the drug development pipeline.

### Intestine–liver models

Drugs that are administered orally are subject to intestinal and hepatic first pass metabolism prior to entering the circulation, which can have a significant effect on the bioavailability, metabolism/biotransformation, and efficacy of drugs.^110^ The intestine affects drug fate by selective absorption as well as metabolism by intestinal epithelial cells and/or microbiome. After absorption, the drug is transported to the liver via the portal vein for further metabolism prior to reaching the systemic circulation through the hepatic vein. Currently, the most common intestine platforms used to study drug adsorption and barrier functionality consist of established intestinal epithelial cell lines (i.e., Caco-2, HT-29) cultured on ECM-coated Transwell^™^ inserts; however, these 2D culture formats fail to recapitulate key intestinal functions, such as villi formation and intestinal CYP activity.^111^ Therefore, the use of more physiologic cell sources, including intestinal 3D organoids derived from stem cell-containing primary intestinal crypts or iPSCs as well as controlled microenvironments, including fluid flow and mechanical activation to induce crypt formation, have become increasingly popular. For example, a 3-chamber PDMS-based “Gut Chip” model contained primary epithelial cells from patient biopsies that were expanded as 3D organoids and then enzymatically fragmented for culture on the top side of a porous PDMS membrane in the center chamber of the device; on the opposite side of the membrane, human intestinal microvascular endothelial cells were cultured.^112^ Two hollow chambers on either side of the center cell-containing chamber allowed for membrane stretching to model peristaltic motion of the small intestine It was found that fluid flow and cyclic stretching facilitated crypt formation with all four lineages of intestinal cells present (enterocytes, goblet, enteroendocrine, and Paneth). Transwell inserts have also been augmented with collagen hydrogels molded in the shape of villi and crypts as a basis for primary intestine cell culture;^113^ the crypt formations maintained stemness/proliferation capabilities while the villi structures contained mature enterocytes/non-proliferating cells as *in vivo*.

Recently, researchers have begun connecting intestinal platforms with liver platforms to model first pass drug metabolism and intestine-liver interactions in physiology and disease. For example, a two-layer microfluidic device was fabricated to allow for the culture of intestine (Caco2) and liver cells in two separate compartments with close interaction to facilitate crosstalk;^114^ both the gut and liver compartments contributed to the metabolism of model compound, apigenin. In another device modeling the small intestine–liver interaction, several physiologic parameters were considered, including shear stress, volume ratios of each organ, and flow rate through the modeled hepatic vein and artery.^115^ In a third device, immortalized primary human intestinal epithelial cells, cultured on polycarbonate membranes, and HepG2 C3A liver cells, cultured on 3D nylon scaffolds, were placed in a 2-compartment chip with gravity-driven perfusion (i.e., on a rocker); the intestinal cells differentiated into major cell types found in native human intestinal epithelium and showed TEER values similar to the native gut, while the HepG2 C3A cells displayed liver functions for 14 days and had higher drug-induced CYP activities in the 2-compartment device with the intestinal cells than in a single compartment on their own.^116^ Finally, quantitative pharmacokinetic studies were conducted utilizing diclofenac and hydrocortisone as model compounds in the multi-MPS platform connecting liver and gut compartments through fluid flow;^117^ key parameters, including intestinal permeability and metabolic clearance of the evaluated compounds, were supported to similar levels as predicted via computational simulations.

The gut–liver interactions are also central for diseases, such as alcoholic fatty liver disease and NAFLD. In a layered device, fatty acids introduced onto the apical side of the intestinal layer were found to be absorbed and subsequently stored by hepatocytes, as assessed via lipid accumulation [Fig. 4(c)].^118^ The device was also able to model a 57% reduction in TEER value after tumor necrosis factor alpha treatment in the intestinal compartment as well as a 30% increase in lipid accumulation in the hepatic compartment, an increase that was not detected in individual tissue type controls. Finally, another device was utilized to assess ethanol-induced hyper-permeability and hepatic stromal injury; fibrotic markers and lipid accumulation increased in coupled devices relative to single tissue controls.^119^

### Heart–liver models

In addition to the liver, the heart is another major organ of interest with respect to drug toxicity concerns as well as serious complications including arrhythmias and torsades de pointes.^120^ Specifically, drug metabolites from the liver can influence the heart and lead to the aforementioned complications.^121^ Microfluidic devices offer the ability to systematically evaluate liver–heart interactions arising from secondary metabolite circulation and off-target interactions. For example, microfluidic systems have been created to model the liver–heart–vasculature interaction utilizing iPSC-derived cells.^122^ In this device, interlocking chambers were fabricated allowing for individual system culture, including liver microtissues, mechanically stretched heart microtissues, and 3D vasculature, all connected by fluid flow. This device exemplifies the ability to combine multiple organ systems through the use of a single iPSC donor with a singular media flow, which can be useful for evaluating multi-organ interactions.

In another device also utilizing iPSC-derived cell sources, a liver microfluidic device was fabricated to mimic a single sinusoid;^123^ this device was comprised of two rectilinear chambers, one for 3D hepatocyte culture and the other for fluid flow, separated by a polyethylene terephthalate membrane with 3 *μ*m pores to mimic the LSEC barrier. For the cardiac device, a center cell chamber containing cardiomyocytes was sandwiched between two adjacent media channels and arrays of connecting microchannels to recapitulate vasculature and nutrient exchange; this device also allowed for cardiomyocyte alignment, junction formation, and *in situ* quantification of spontaneous beating. For a proof-of-concept drug study in this coupled liver–cardiac device, inhibition of cisapride (drug to treat heartburn) metabolism by the liver device via a CYP3A4 inhibitor, ketoconazole, led to a significantly prolonged action potential duration in the coupled cardiac microfluidic system.^124^ In another platform, a PHH chamber was connected fluidically to an iPSC-derived cardiomyocyte chamber with integrated microelectrode array and cantilever chips to evaluate the electrical and mechanical properties of the cardiomyocytes [Fig. 4(d)];^125^ when cardiotoxic compounds (e.g., cyclophosphamide, a chemotherapy drug that is metabolized by the liver into a cardiotoxic by-product) were tested in this device, iPSC-derived cardiomyocyte beating rate and viability decreased without affecting PHH functions.

### BOC devices

Success of single- and dual-organ chip-based platforms has spurred the creation of platforms with additional tissue compartments, namely, BOC platforms that aim to recapitulate several organ model systems within a human-like microenvironment to study *in vivo*-like drug pharmacokinetics and pharmacodynamics (PKPD).^126^ Several BOC devices allow for end-user customization of organ systems using a plug-and-play configuration of microfluidic compartments. Individual organ systems can first be established and then combined into an integrated system. For example, a hanging drop array-based device was fabricated to allow cells of different organs to first be seeded into individual columns of a 6 × 4 array of hanging drops;^127^ the microfluidic network could then be reconfigured to switch perfusion through each row, simulating organ–organ interactions. Another platform, entitled the multi-MPS, has been utilized to construct 4-, 7-, and 10-MPS organ systems utilizing advanced recirculation architectures to better mimic blood flow and differential fluid distribution from organ to organ [Fig. 4(e)].^128^ This platform requires an array of individually modulated micropumps and microchannels integrated into the base of the device. A system of micropumps driven by electromagnetic actuation allowed for the integration of up to 10 individual Transwell insert-based organ systems, currently allowing for single pass and recirculating media flow.^129^ While these devices are useful in terms of ease of configurability and user input, they do not allow for control of relevant fluid-to-tissue ratios. For example, in the multi-MPS platform the volume of fluid that is circulated is 13-fold of the volume of fluid in the tissue chambers, a significant variation from *in vivo* ratios,^128^ which could be detrimental to clinically relevant modeling of PKPD as metabolites may be drastically diluted, leading to sub- or non-physiologic responses.

In contrast to the reconfigurable systems, non-configurable platforms support a set number of organ systems and allow for better control over fluid volumes and/or native tissue architecture. For example, a multi-channel 3D microfluidic cell culture system combined individual liver, kidney, lung, and adipose cell culture compartments for drug toxicity assessment.^130^ Other devices aim to mimic blood flow through controlled pulsatile medium perfusion at relevant tissue-to-liquid ratios. For instance, compartments with a reconstructed 3D small intestine for absorption of orally administered compounds, a skin biopsy to model dermal substance absorption, liver for metabolism, and kidney compartment to support metabolite excretion were combined to create a stable human-on-a-chip platform for an extended 28-day culture period [Fig. 4(f)].^131^ The device design incorporated injection ports at different points to study the behavior of different drugs based on administration routes.

## FUTURE OUTLOOK AND CONCLUSIONS

*In vitro* liver models must be scalable to support different throughput needs within various stages of drug development, support phenotypic stability of hepatocytes and liver NPCs at levels close (>75%) to those observed *in vivo* for 1–4 weeks to enable multiple short- and long-term applications, be easy to use by the end-user, and provide reproducible outcomes with different cell and device/plate lots. As advanced 2D and 3D liver platforms have both been utilized for a myriad of applications in the drug development pipeline, both have benefits and drawbacks. For example, 3D cultures (e.g., spheroids, organoids, and bioprinted tissues) provide more physiologic stiffness, architecture/compartments, and cell–cell/cell–ECM interactions as compared to coplanar (monolayer) models on stiff tissue culture polystyrene. However, difficulties with adaptation to high content imaging (HCI) and increased complexity of drug diffusion kinetics make the use of 3D cultures more difficult for HTS. Comparatively, advanced 2D culture platforms, like the MPCC, allow for HCI^132^ and direct access of cells to drugs and other types of therapeutics (e.g., nanoparticles); MPCCs have also been adapted to 384-well plates for HTS applications.^30^ Nonetheless, both spheroids/organoids and MPCCs have been validated to maintain long-term (>4 weeks) liver functions and allow for the inclusion of different liver NPC populations to assess drug effects and model liver physiology and disease as necessary. Ultimately, however, a 3D printed liver tissue with hepatic, vascular, and biliary compartments will be necessary to evaluate drug effects on all liver compartments and how drug effects on one compartment affect the other compartments. For microfluidic devices, single-organ or multi-organ throughput is often compromised and cost increases due to the need for dedicated perfusion equipment; however, physiological complexity can be modeled closer to that observed *in vivo* (e.g., fluid shear stress, factor gradients, inter-tissue crosstalk), which affords the opportunity to test hypotheses not possible with static cultures (e.g., zonal drug toxicity, drug metabolism in liver causing toxicity to other organs, first pass metabolism by intestine followed by metabolism by liver). While *in situ* and real-time monitoring of protein or metabolite formation, oxygen consumption, and/or cell barrier integrity are being implemented within some microfluidic platforms via electrochemical sensors,^109,133^ more widespread incorporation of such approaches will allow for rapid detection of cell responses to drugs for a high-throughput drug screening campaign. We anticipate that biopharmaceutical companies will continue to utilize liver and BOC models of different technological and physiological complexities depending on throughput needs and hypotheses being posed; such is an advantage of the availability of different types of *in vitro* models. We summarize the key features and applications of representative liver and multi-tissue platforms in Table I.

**TABLE I.** Engineered platforms with applications in drug development.

Model	Organ	Cells	Unique contribution	Validated applications
MPCC—HepatoPac (BioIVT)^7^	Liver	PHHs, iHeps, 3T3-J2 murine embryonic fibroblasts, LSECs, HSCs, and KCs	Maintains highly functional PHHs for up to 10 weeks and liver NPCs for up to 4 weeks in monolayer format and uses standard multi-well plates (up to 384-well format)	Drug toxicity, drug clearance, DDI, infection with HBV/HCV/malaria, and NAFLD modeling
3D Insight^™^ spheroids (InSphero)^67^	Liver	PHHs, LSECs, KCs, and HSCs	Maintains relatively stable gene and protein expression for over 35 days of 3D culture and requires fewer cells than other model systems	Drug toxicity and NAFLD modeling
Spheroids formed in ultra-low attachment plates (HepaPredict)^70^	Liver	PHHs, KCs, HSCs, and biliary cells	Long-term 3D model and allows for spheroid size tuning	Drug toxicity, DDI, and NAFLD modeling
Liver-chip (Emulate)^94^	Liver	PHHs, LSEC, KCs, and HSCs	Mimics liver architecture with porous membrane separating hepatocytes from NPCs, and two channels allow perfusion of PHHs and LSECs with different flow rates	Drug toxicity
LAMPs (NortisBio)^99^	Liver	PHHs, HMVECs or LSECs, THP-1, and HSCs	Oxygen control via variable medium perfusion rates to model zones 1 and 3 liver phenotypes	Zone-specific drug toxicity
PREDICT-96 (Draper)^107^	Liver	PHHs	One plate contains 96 two-channel microfluidic devices with a pump array	Drug toxicity
OrganoPlate (Mimetas)^108^	Liver	iHeps, HMVECs, and THP-1	96-microfluidic devices in one chip allows for high-throughput screening, and compatible with commercial fluid handlers	Drug toxicity
InLiver-OC (Istituto Italiano di Tecnologia)^119^	Intestine, liver	Intestinal myofibroblasts, Caco-2, and HepG2	Modeling of first pass metabolism allowing for sampling after each organ compartment and allows for alcohol to be included in system without evaporation	Alcoholic fatty liver disease modeling
Gastrointestinal (GI) tract–liver system (Cornell)^116^	Intestine, liver	Immortalized human intestinal epithelial cells, intestinal myofibroblasts, and HepG2 C3A	Retains major cell types of intestinal epithelium, tight junction formation in the intestinal layer of device with sustained TEER permeability values, and maintenance of intestine and liver CYP activity	Intestinal absorption characteristics
Heart–liver MPS (Hesperos)^125^	Heart, liver	iPSC-derived cardiomyocytes and PHHs	Real-time monitoring of cardiomyocyte electrical and mechanical changes, and allows assessment of the effect of liver drug metabolism on cardiomyocytes	Drug metabolism, toxicity, and drug effects on electrical parameters of cardiomyocytes
Multi-organ chip (UC-Berkeley)^124^	Heart, liver	iPSC-derived cardiomyocytes and PHHs	All cell types derived from the same iPSC line	Drug uptake/efflux, metabolism, toxicity, and drug effects on electrical parameters of cardiomyocytes
MPS (Draper)^109^	Liver, intestine, kidney	PHHs, endothelial cells, intestinal epithelial cells, and renal proximal tubule epithelial cells	96 microfluidic devices in a plate format, integrated pumps, and TEER measurement system in plate lid, and embedded oxygen sensors	Shear stress effects on cell phenotype, cell layer permeability, and renal transport
MPS (Draper)^129^	Up to 12 tissue types	Various	Reconfigurable per end user's requirement and utilizes electromagnetic actuation	Multi-organ toxicity
MPS (CN-Bio)^128^	Up to 4, 7, or 10 tissue types	Various	Reconfigurable per user's requirements and customized chambers for physiologic fluid flow	Multi-organ toxicity, drug distribution

While isolated primary hepatocytes and NPCs are considered most physiologically relevant to build human liver models, these cells are in limited supply due to organ shortages and thus are not always amenable to HTS of very large compound libraries. That being said, efforts are under way to harness the *in vivo*-like proliferative potential of PHHs at least and utilize the expanded cells in advanced culture systems for drug screening;^134^ however, further differentiation of the expanded (and immature following proliferation) PHHs is needed. Regardless, donated organ shortage makes it difficult to screen for genetic determinants of DILI in primary cells, a limitation that can be mitigated via iPSC technology. Importantly, iPSCs can be derived from large patient populations with specific mutations in DMEs and/or can be edited in their genetic makeup via techniques, such as CRISPR to model specific genotypes. While iPSC-derived liver cells will most likely revolutionize drug screening in the future, much more research is currently needed to determine the microenvironmental conditions and gene circuits that can produce reproducibly differentiated iPSC-derived human liver-like cell types with nearly physiologic, adult-like, phenotypic functions. The combination of synthetic biology and advanced culture techniques is highly promising to accelerate such a goal.^135^ Even so, before iPSC-derived human liver-like cells can be routinely used for HTS, the cost of differentiation will need to be reduced to make the overall cost for screening comparable to that of commercially available primary human liver cells. Additionally, to evaluate the role of genetics on drug outcomes, both hepatocyte-like and NPC-like cells in the liver model will need to be generated from the same iPSC source; toward that end, protocols to differentiate iPSCs into liver NPC-like cell types need further improvement. Finally, donor-matched adaptive immune cells will ultimately need to be included into iPSC-derived human liver models with sufficient numbers of genetically diverse donors to address the idiosyncratic DILI that tends to only show up once a drug enters the broader marketplace and is taken by millions of patients; in that sense, iPSC technology is well poised to make a significant impact for the detection of both dose-dependent and idiosyncratic DILI outcomes.

Phenotypic functions of *in vitro* liver models over time need to be thoroughly appraised relative to freshly isolated tissue and/or isolated liver cell types. Global transcriptomics can provide an initial snapshot of how the *in vitro* liver model compares to native liver tissue, but such needs to be complemented with functional analysis. However, since it is impractical to assay 500+ liver functions for any application, major categories of liver functions can be evaluated via prototypical markers as we have summarized in Table II. For example, albumin production corresponds to hepatocytes' transcription, translation, processing, and export functionality, while urea secretion can be used to evaluate mitochondrial activity, biochemical synthesis, and ammonia detoxification.^136^ Both biomarkers can be transiently appraised from collected supernatants, as opposed to assessment via cell lysis (i.e., destructive assays), and are widely utilized as surrogate markers to assess hepatocyte health.^7^ Comparatively, maturation should be considered for iPSC-derived hepatocyte cell sources, for example, through evaluation of the albumin to alpha-fetoprotein and CYP3A4 to CYP3A7 ratios (i.e., increasing ratios indicate higher maturity).

**TABLE II.** Major functions of different liver cell types that can be measured within engineered devices.

Hepatocyte functions (method)	LSEC functions (method)	KC functions (method)	HSC functions (method)
• *Protein synthesis*: albumin, transferrin, and alpha-1 antitrypsin (AAT) secretions [enzyme-linked immunosorbant assay (ELISA)]	• Factor VIII secretion (ELISA)	• Tumor necrosis factor alpha and interleukin-6 secretion following 24-h stimulation with lipopolysaccharide (ELISA)	• Lipid/vitamin A droplets (fluorescent stain)—indicates quiescent phenotype
• *Ammonia detoxification*: urea secretion (colorimetric kit)	• *Cell surface markers:* CD31 and CD32b/SE-1 (immunostaining)	• Phagocytosis (fluorescent bioparticle assay)	• *Protein markers* Desmin, α-smooth muscle actin (α-SMA) (immunostaining)—high α-SMA is indicative of an activated or myofibroblastic HSC phenotype
• *Drug metabolism enzyme activities*: CYP1A2, 2A6, 2B6, 2D6, 2C8, 2C9, 2C19, 3A4, UGT, and SULT (fluorescent and luminescent metabolites of substrates and detection of substrate metabolites via LC-MS/MS)	• Fenestrae in sinusoidal endothelial cells (scanning electron microscopy)	• *Surface markers:* CD68 and CD14 (immunostaining)	
• *Carbohydrate metabolism*: *de novo* glucose secretion (gluconeogenesis) ± hormones (insulin and glucagon) (colorimetric kit)			
• *Lipid metabolism*: neutral lipid accumulation (fluorescent stain)			
• *Transporter functions*: OATP1B1/1B3/2B1, OCT1, OAT2/7, NTCP, MRP2/3/4/6, BSEP, P-gp, BCRP, and MATE1 (fluorescent drug and bile analogs, radiolabeled drugs)			
• *Toxicity*: release of alanine aminotransferase (ALT) and aspartate aminotransferase (AST) in supernatants			

To assess DILI, the use of general toxicity markers, such as ATP or lactate dehydrogenase release from cells, is common;^137^ however, these markers are not able to distinguish hepatotoxicity from toxicity to different liver cell types within a coculture model. Therefore, cell-specific markers are needed and often can be more sensitive for detecting DILI at more pharmacologically relevant drug concentrations before the onset of overt drug toxicity; indeed, we and others have shown that albumin and urea secretions are significantly downregulated well before detectable loss of ATP.^6,43,138^ Similarly, changes in the expression of stress and metabolic pathways precedes overt drug toxicity and can be used to classify compounds with increased DILI risk.^43,139^ We anticipate that early markers of DILI outcomes will become more common moving forward.

Often, liver platforms are validated against different drug sets for sensitivity and specificity for DILI detection, which makes it difficult to compare platforms for utility across different applications. One strategy that may enable better comparisons across different liver models is to select and classify drugs based on the Liver Toxicity Knowledge Base maintained by the FDA's National Center of Toxicology Research, which allows for classification of drugs in various categories, such as severe clinical DILI, high clinical DILI concern, low clinical DILI concern, enzyme elevations in the clinic, and no DILI.^140^ When choosing a drug set for platform validation, drugs from different classes and major mechanisms of action implicated in DILI (e.g., mitochondrial disruption, reactive metabolites, disruption of lipid and protein metabolism, transporter inhibition, and cholestasis) should be selected. Toward that end, there is a need for a clear definition of the key characteristics of DILI, similar to what has been done for carcinogenesis caused by compounds.^141^ For drug concentrations, MOS of 50–100× are common for *in vitro* testing and still provide high specificities (>85%), though DILI can be detected at more clinically relevant drug concentrations with longer/repeat incubations on metabolically competent liver models and with use of endpoints that can detect adverse effects earlier than overt toxicity as discussed above. Finally, the validation of advanced liver models needs to be carried out across multiple sites, including the biopharmaceutical industry, to assess the reproducibility and robustness across multiple cell lots, device/plate batches, and operators. Such types of multicenter validations are beginning to emerge but need to be practiced more broadly.

Even though human liver models and multi-organ devices containing liver models need further refinement, validation, and standardization to become routine fixtures within the drug development pipeline, their impact is already apparent as demonstrated via relevant examples above. We anticipate that as humanity tries to eradicate existing diseases and navigate the emergence of new diseases, it will be critical to develop safe and efficacious therapies for patients much faster and more cost effectively than in the past, while reducing animal usage to the extent possible since animals often do not adequately model human responses to drugs, especially for the liver. Toward that end, *in vitro* human liver models and their combinations with other organ models will be indispensable for continued successes of the biopharmaceutical enterprise.

## Data Availability

Data sharing is not applicable to this article as no new data were created or analyzed in this study.
